# Association between Four Dietary Patterns and the Risk of Periodontal Diseases: A Systematic Review and Meta-Analysis

**DOI:** 10.3390/nu14204362

**Published:** 2022-10-18

**Authors:** Jaehun Jeong, Hyung-Sik Kim, Dongjun Lee, Kihun Kim, Yun-Hak Kim

**Affiliations:** 1 School of Dentistry, Pusan National University, Yangsan 50612, Korea; 2 Department of Oral Biochemistry, Dental and Life Science Institute, School of Dentistry, Pusan National University, Yangsan 50612, Korea; 3 Department of Convergence Medicine, School of Medicine, Pusan National University, Yangsan 50612, Korea; 4 Department of Occupational and Environmental Medicine, Kosin University Gospel Hospital, Busan 49267, Korea; 5 Department of Biomedical Informatics, School of Medicine, Pusan National University, Yangsan 50612, Korea; 6 Department of Anatomy, School of Medicine, Pusan National University, Yangsan 50612, Korea; 7 Research Institute for Convergence of Biomedical Science and Technology, Pusan National University Yangsan Hospital, Yangsan 50612, Korea

**Keywords:** periodontal disease, sugar, dairy product, western diet, vitamin C

## Abstract

Background: Several dietary patterns are reported as risk factors for several chronic diseases including oral diseases. However, thus far, there has been no comprehensive quantitative analysis of nutrition and periodontal diseases. Methods: This systematic review was conducted according to the PRISMA guidelines. Cohort, case–control, and cross-sectional studies were eligible for inclusion in this study. The Newcastle–Ottawa scale was used to qualitatively assess the risk of bias in the included studies. The number of samples was used for odds ratio calculation, followed by the unadjusted value and 95% confidence interval. Results: Nine papers were included for the systematic review and meta-analysis. The results of screening for database search records showed that four diet patterns (western diet, dairy product intake, sugar intake, and vitamin C intake) have enough data for meta-analysis. The risk of periodontal disease in the western-diet group and the lowest dairy product intake group was 1.05 (0.51–2.13) and 1.28 (0.89–1.84), respectively. The risk of periodontal disease in the highest sugar intake group and the lowest vitamin C intake group was 1.52 (0.79–2.91) and 1.15 (1.08–1.23), respectively. Conclusions: With aging of the population globally, the prevalence of periodontal disease increases, and the associated cost also increases. Though this study, we found foods related to the risk of periodontal disease, and we are confident that it will contribute to lowering the incidence of the disease.

## 1. Introduction

Periodontal disease is an oral, chronic infectious, and inflammatory disease that can damage the supportive tissues surrounding the teeth and consequently cause tooth loss [1,2]. The prevalence of periodontitis was 46% in 2010, and 3.9 billion people worldwide were reported to have periodontal disease [3]. According to the 2016 Global Burden of Disease Study, periodontal disease is the 11th most prevalent disease worldwide. Additionally, the prevalence of periodontal disease increased by 25.4% from 2005 to 2015, with an estimated global cost of 442 billion USD [4]. Periodontal disease has become an important issue with aging globally, and continuous research has been conducted on this topic [5]. As the importance of the relationship between periodontal disease and systemic inflammatory diseases increases, the importance of research on risk factors for periodontal diseases also increases [6]. The possibility of an association between periodontal disease and various systemic diseases (Alzheimer’s disease, respiratory disease, diabetes, pneumonia and some malignancies) has been suggested, but it is not clearly elucidated by the influence of confounding variables [6,7].

Nutritional intake and dietary patterns have been studied as risk factors for several chronic diseases including oral diseases [2,8,9]. According to a recently published literature reviews, it was reported that there was an inverse correlation with periodontitis among intake of omega-3 fatty acids, vitamins, C, beta-carotene, fibre, calcium, dairy products, zinc, polyphenols, and fruits and vegetables [10,11,12,13]. In addition, the potential for periodontitis treatment of probiotics and prebiotics intake was suggested [14]. On the other hand, dietary habits that are considered unhealthy, such as high sugar, high saturated fat, low unsaturated fat, and low fibre, were suggested to increase the risk of periodontitis [10,15]. The association of vitamin D, E, K and magnesium intake with periodontitis remains unclear [10].

As mentioned above, several review papers and systematic literature reviews have been reported on the association between various dietary habits and periodontitis, but a meta-analysis performed by quantifying the related papers has not been published. Therefore, the aim of this paper is to conduct a systematic literature review and meta-analysis on the association between specific dietary habits and periodontitis.

## 2. Methods

### 2.1. Protocol Registration

PRISMA guidelines were followed in conducting this systematic review [16]. A protocol for the study was registered with PROSPERO (CRD42021235226).

### 2.2. Search Strategy

The search was performed on 29 January 2021, using Embase and Medline. The search words were discussed by all authors. The detailed search terms are as follows: (fiber:ab,ti OR food:ab,ti OR diet:ab,ti OR intake:ab,ti OR uptake:ab,ti OR ingestion:ab,ti OR consupmtion:ab,ti OR habit:ab,ti OR pattern:ab,ti OR fruit$:ab,ti OR grain$:ab,ti OR vegetable$:ab,ti OR bean$:ab,ti OR legume$:ab,ti OR nut$:ab,ti OR seed$:ab,ti) AND (periodontitis:ab,ti OR ‘periodontal disease’:ab,ti OR ‘periodontal inflammation’:ab,ti OR ‘gum disease’:ab,ti OR ‘gum inflammation’:ab,ti OR gingivitis:ab,ti OR parodontitis:ab,ti OR paradentitis:ab,ti) AND (risk:ab,ti OR ratio:ab,ti OR prevalence:ab,ti OR incidence:ab,ti OR morbidity:ab,ti OR odds:ab,ti OR hazard:ab,ti). We did not have restrictions by language or year of publication.

## 3. Eligibility Criteria

We defined the PICO question as follows: Does the group with a particular dietary pattern increase the risk of periodontitis compared to the group without it? In this study, a term that includes various diets and nutritional intake such as western diet, sugar and vitamin C intake, and dairy product was defined as the term ‘dietary pattern’. The results of screening for database search records showed that these four diet patterns have enough data for meta-analysis. We included studies that presented the number of samples or effect measures. The western diet is defined as a diet that contains a large amount of saturated fat, refined carbohydrates, and salt and has a low proportion of fruits and vegetables [17]. Dairy product Includes total dairy product intake as well as intake of milk, yogurt, cheese and lactic acid food. Sugar consumption includes information on sugar added to both food and drink. Vitamin C intake was investigated for vitamin C added to the diet. Papers that did not provide results on the association between dietary pattern and periodontal disease or did not match the diet pattern we categorized were excluded. Periodontitis was defined as periodontal pocket depth (PPD) ≥ 4 mm or clinical attachment level (CAL) ≥ 1 mm or community periodontal index (CPI) ≥ 3 [18,19]. We excluded the papers that did not meet the periodontitis criteria. Cohort, case-control, and cross-sectional studies were eligible for inclusion in this study. Studies that evaluated a cohort of patients with specific conditions (e.g., cigarette smoking) were not subject to review. Only human studies were included, and conference papers and review papers were excluded from analysis. We did not have restrictions by language or year of publication.

### 3.1. Study Selection

The titles and abstracts of each study were reviewed independently by two authors (JJ and KK). For inclusion, the same authors reviewed full-text articles. Disagreements were resolved through discussion.

### 3.2. Data Extraction

The following variables were extracted during the screening phase: title, abstract, journal, author name, publication year, and publication type. We extracted additional information from the full-text assessment, such as the study design, WHO region, number of samples, type of diet, diagnostic criteria, race, and age.

### 3.3. Assessment of Risk of Bias

Qualitative risk of bias assessments were conducted for the cohorts and case-control studies based on the Newcastle-Ottawa scale [20]. We used an adapted version of the Newcastle–Ottawa scale for cross-sectional studies [21]. The two authors (JJ and KK) independently assessed the risk of bias of the included studies. Discrepancies in the assessment were resolved through discussion by the same authors. In accordance with the Agency for Healthcare Research and Quality (AHRQ) standard, cohort and case-control studies were rated good, fair, and poor. Cross-sectional studies were evaluated as ‘very good’, ‘good’, ‘satisfactory’, and ‘unsatisfactory’ [22].

### 3.4. Data Synthesis

Odds ratio and 95% confidence interval was extracted by a 2 × 2 contingency table of exposures and outcomes. We performed subgroup analysis by classifying the diet patterns into 4 categories; western diet, dairy product, sugar, and Vitamin C. Forest plots were drawn to clearly visualise the synthesised risk. Evaluation of the effect measures’ heterogeneity was conducted using the classification of I^2^ statistics [23]. In case the heterogeneity exceeds 50%, we used the random-effects method; otherwise, we used the fixed-effects method. The results were synthesized using Review Manager 5.4 software (version 5.4.1, Cochrane Training, UK).

## 4. Results

### 4.1. Study Selection

**Figure 1:**
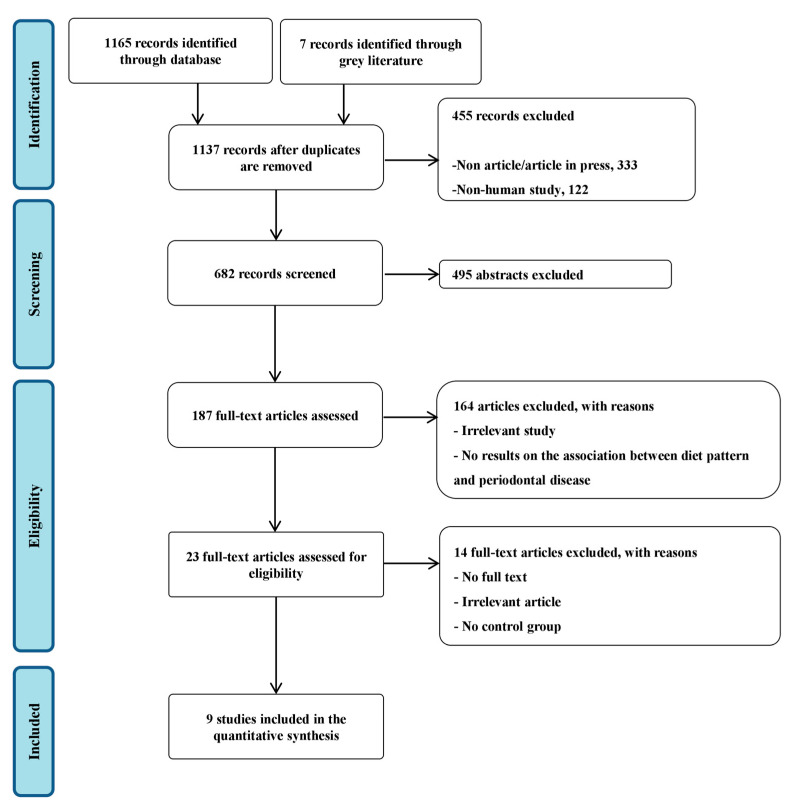
PRISMA flowchart of the study selection process.

We identified and screened 682 potentially relevant articles (including overlapping publications across the two databases); 495 articles were excluded on the basis of the title and abstract and 187 articles were further searched for a more detailed assessment. After further evaluation of the full articles, 168 were excluded because they had study designs other than the pertinent one, or no results on the association between diet pattern and periodontal disease were presented. Finally, 9 studies were included in the systematic review and meta-analysis (Figure 1). The characteristics of the included studies are summarised in Table 1.

### 4.2. Western Diet

**Figure 2:**
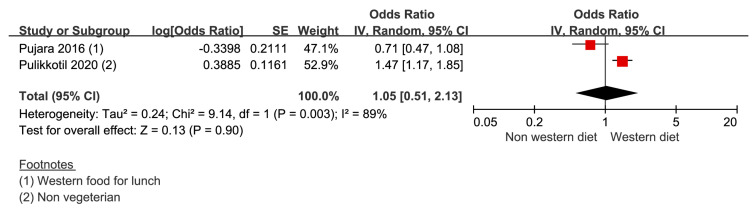
Risk of periodontal disease with the western diet [24,25]. -red color: weight for overall effect; -black color: 95% confidence interval.

Two studies provided two data sets were included to derive the result. The risk of periodontal disease in the western-diet group was 1.05 (0.51–2.13) compared to that in the non-western diet group (Figure 2). The heterogeneity was 89%.

### 4.3. Dairy Product

**Figure 3:**
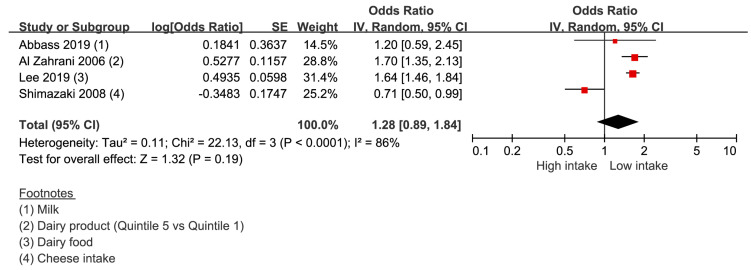
Risk of periodontal disease with dairy products [26,27,28,29]. -red color: weight for overall effect; -black color: 95% confidence interval.

Four studies provided four data sets were included to derive the result. The risk of periodontal disease in the lowest dairy product intake group was 1.28 (0.89–1.84) compared with that in the highest dairy product intake group (Figure 3). The heterogeneity was 86%.

### 4.4. Sugar

**Figure 4:**
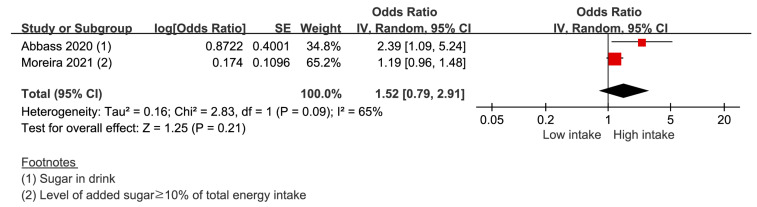
Risk of periodontal disease with the high-sugar diet [26,30]. -red color: weight for overall effect; -black color: 95% confidence interval.

Two studies provided two data sets were included to derive the result. The risk of periodontal disease in the highest sugar intake group was 1.52 (0.79–2.91) compared with that in the lowest sugar intake group (Figure 4). The heterogeneity was 65%.

### 4.5. Vitamin C

**Figure 5:**
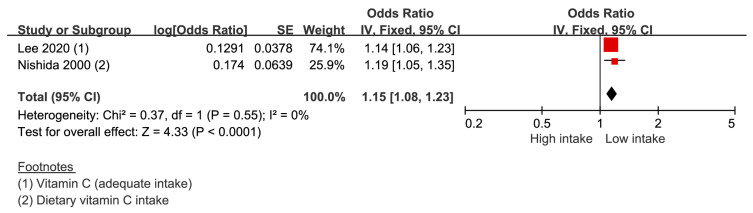
Risk of periodontal disease with vitamin C intake [31,32]. -red color: weight for overall effect; -black color: 95% confidence interval.

Two studies provided two data sets were included to derive the result. The risk of periodontal disease in the lowest vitamin C intake group was 1.15 (1.08–1.23) compared with that in the highest vitamin C intake group (Figure 5). The heterogeneity was 0%.

### 4.6. Risk of Bias within Studies

One cohort study was rated good. One case-control study was rated Fair. Out of 13 cross-sectional studies, 8 were evaluated as very good, 3 as good, and 2 as satisfactory. Detailed evaluation results are presented in Supplementary Materials.

## 5. Discussion

In this systematic review and meta-analysis of observational studies, we found that certain nutritional and dietary patterns significantly affected the prevalence of periodontal disease. Western diet, dairy products, and high sugar intake were not statistically significant with periodontitis, and vitamin C low intake was positively associated with risk of periodontitis. Because poor periodontal health may cause systemic diseases such as cardiovascular diseases, cancers, and diabetes, oral hygiene-related factors should be studied in detail.

Western diet, which includes processed foods, red meat, snacks, and soft drinks, is a well-studied risk factor for inflammatory diseases and many types of malignancies [33,34]. Long-term intake of a western diet can cause health problems by promoting weight gain, pathophysiological changes, and activation of the immune system [33]. Chapple et al. (2009) suggested that consumption of highly refined and processed foods causes postprandial inflammation induced by oxidative stress [35]. Genko et al. (2005) argued that free fatty acid intake increased obesity and glucose intolerance, which could increase the risk of periodontal infection through the induction of inflammatory cytokines [36]. Martinon et al. (2019) reported that a diet considered to be an unhealthy diet such as high sugar, high saturated fat, and low fibre increases the risk of periodontitis [10]. However, our meta-analysis did not show a significant association. It was thought to be the result of the small number of papers included due to duplicate databases, inability to utilize nutritional intake information, and different definition of periodontitis.

Dairy products (milk, yoghurt, lactic acid food, and cheese) are one of the most studied foods for their association with several diseases. Although there is no association with the risk of all-cause mortality, many epidemiological studies have reported that dairy products have protective effects against periodontal diseases, cancers, and chronic inflammatory diseases, especially cardiometabolic diseases [37,38,39,40,41,42]. There is a hypothesis that probiotics help the production of substances that inhibit periodontopathic bacteria [43]. Other studies have suggested that lactic acid may have a beneficial effect on periodontal health because of their probiotic effects [28]. A recently published review paper suggested evidence for the positive health effects of dairy products although the interpretation is limited due to the analysis of cross-sectional studies [13]. The results of our paper showed a insignificant association, which is assumed due to the lack of included papers, but a follow-up study would be needed on the positive effect of dairy products on periodontitis, which is inferred by biomechanical evidence.

A high-sugar diet contributes to the formation of dental biofilms and, consequently, caries and reduction of pH [44,45]. Hyperglycaemia creates an inflammatory environment through the generation of oxidative stress [44]. A high-sugar diet is a common risk factor for both dental caries and periodontal disease [46]. Hyperglycaemic conditions induce dyslipidaemia and insulin resistance, resulting in metabolic syndrome [47]. Most of these metabolic syndrome-related conditions are reported to be associated with periodontitis [48,49,50]. Systemic inflammation and oxidative stress induced by metabolic dysregulation may be suggested as potential mechanisms [30]. High-sugar consumption could adversely affect bone metabolism by increasing alkaline phosphatase activity and inhibiting calcium and phosphorus homeostasis [51]. In addition, high concentrations of alkaline phosphatase have been reported to cause alveolar bone loss and periodontitis [52,53]. In a systematic literature review on sugar-sweetened beverages and periodontal disease published recently, it was reported that sugar-sweetened beverages may cause periodontitis by increasing periodontal bleeding [15]. Our results did not show significant statistical relevance, but excessive sugar intake should be avoided because it has been proven to be related to systemic diseases [54,55].

We found that vitamin C intake slightly reduced the prevalence of periodontal disease. According to two recently published systematic reviews of the literature, it was reported that low vitamin C intake and low vitamin blood levels increase the risk and progression of periodontitis [11,12]. Our results of vitamin C intake also support these evidence. It’s possible that lower vitamin C intake is a marker for lower diet quality in general (less fruit and vegetables) [56]. Vitamin C supplementation is known to decrease the production of reactive oxygen species and inflammatory reactions [57,58]. In addition, vitamin C is a cofactor necessary for the hydroxylation of proline and lysine, which is essential for maintaining the integrity of connective tissue [59]. However, the effect of mild vitamin C deficiency on periodontal disease remains unclear [59].

Our meta-analysis had several limitations that could have affected the results. First, it was difficult to clearly identify the causal relationship because the majority of the included studies in the meta-analysis had a cross-sectional design. Second, errors in reporting, such as food frequency questionnaires, or uncertainties about the source and composition of food, during the course of the investigation may have affected drawing conclusions. Third, it was impossible to calculate the total number of patients with periodontal disease included in the study because the articles did not present information on the sample size. Finally, three (rather than 2) data sets are often considered the minimum for meta-analysis, but in this study, the number of included studies for each diet pattern was very small.

## 6. Conclusions

In this study, we analysed the relationship between various food patterns and the prevalence of periodontal diseases through a comprehensive analysis of dietary habits. Overall, there were no deviations from the conventional common sense. The diet suggested in our study was considered to be similar to various diets used for the prevention of metabolic disease. With aging of the population globally, the prevalence of periodontal disease increases, and the associated cost also increases. Therefore, by studying this issue further and controlling the intake of foods that appear to be a risk factor, the risk of periodontitis could be reduced.

## Figures and Tables

**Table 1:** Characteristics of the included studies.

Author, Year	Study Design	Type of Diet	Specific Diet Pattern	Degree	WHO Region	Number of Samples	Age, Years	Exposure Assessment	Periodontitis Criteria *
Pulikkotil, 2020 [24]	Case-control	Western diet	Non vegetarian diet	2-quantile	India	Cases 604 Controls 604	35–44	interviewed with a piloted questionnaire.	CPI ≥ 4 mm
Pujara, 2016 [25]	Cross-sectional	Western diet	Snack and dessert	Never/sometimes/often/very often	India	800	19–25	Self-administered structured questionnaire	CAL ≥ 4 mm and PPD ≥ 5 mm;
Abbass, 2019 [26]	Cross-sectional	Dairy product and sugar	Milk/milk product/candy/sugars in drinks	≤2 times/week/3–6 times/week/1–6 times/day	Egypt	343	18–74	Questionnaire (not otherwise specified)	CAL ≥ 1 mm
Lee, 2019 [27]	Cross-sectional	Dairy product	Milk and yogurt	Never/0–1/1–3/3–7/≥7 Servings/Week	Korea	9798	≥30	Semi-quantitative food frequency questionnaire	CPI ≥ 3
Shimazaki, 2008 [28]	Cross-sectional	Dairy product	Cheese/lactic acid food/other dairy product	0/0.1–3.4/3.5–6.9/≥7.0 g/day	Japan	942	40–79	Semi-quantitative food frequency method	PPD ≥ 4 mm
Al-Zahrani, 2006 [29]	Cross-sectional	Dairy product	Milk and milk products.	Quintiles	U.S.A.	12,764	≥18	24-h dietary recall household interview	CAL ≥ 3 mm and PPD ≥ 4 mm
Moreira, 2021 [30]	Cross-sectional	Sugar	Added sugar	<10%/≥10% of total energy intake	Brazil	2515	18–19	Food frequency questionnaire (FFQ)	CAL ≥ 4 mm and PPD ≥ 4 mm
Lee, 2020 [31]	Cross-sectional	Vitamin C	Daily vit C intake	Adequate intake (75 mg ≤ intake < 1999 mg/d)/Inadequate(intake < 75 mg/d; over UL: intake ≥ 2000 mg/d)	Korea	12,750	≥19	24-h dietary recall method by food frequency questionnaire (FFQ)	CPI 3–4
Nishida, 2000 [32]	Cross-sectional	Vitamin C	Daily vit C intake	Quintiles	U.S.A.	12,419	≥20	24-h recall conducted in mobile examination centre	CAL ≥ 1.5 mm

* CAL: clinical attachment level, CPI: community periodontal index, PPD: periodontal pocket depth.

## Data Availability

Not applicable.

## Supplementary Materials

The following supporting information can be downloaded at: https://www.mdpi.com/article/10.3390/nu14204362/s1.
