# Supplementary material for: Association between Four Dietary Patterns and the Risk of Periodontal Diseases: A Systematic Review and Meta-Analysis
Source: Nutrients. 2022 Oct 18;14(20):4362. doi: 10.3390/nu14204362 (PMC9611912; doi:10.3390/nu14204362)
Supplement: Supplementary file 1 [file nutrients-14-04362-s001.zip › Supplementary materials.pdf]

# Supplementary text

**Newcastle–Ottawa Scale - adapted for cross-sectional studies [1]**

**Selection:** (Maximum 5 stars)

1) Representativeness of the sample
a) Truly representative of the average in the target country or region* (all subjects or random sampling)
b) Somewhat representative of the average in the target country or region* (non-random sampling)
c) Selected group of users
d) No description of the sampling strategy

2) Sample size
a) Pre-determined and calculated*
b) Not pre-determined or calculated.

3) Non-respondents
a) Comparability between respondents' and non-respondents' characteristics is established, and the response rate is satisfactory* (70% response rate).
b) The response rate is unsatisfactory, or the comparability between respondents and non-respondents is unsatisfactory (70% response rate).
c) No description of the response rate or the characteristics of the responders and the non-responders.

4) Ascertainment of the exposure (risk factor)
a) Validated measurement tool**
b) Non-validated measurement tool, but the tool is available or described*
c) No description of the measurement tool

**Comparability:** (Maximum 2 stars)

1) The subjects in different outcome groups are comparable based on the study design or analysis. Confounding factors are controlled for.
a) The study controls for toothbrushing (select one)*
b) The study controls for other demographic factors*

**Outcome:** (Maximum 3 stars)

1) Assessment of the outcome
a) Independent blind assessment**
b) Record linkage**
c) Self-report*
d) No description

2) Statistical test:
a) The statistical test used to analyse data is clearly described and appropriate, and measurement of the association is presented, including confidence intervals and the probability level (p value)*
b) The statistical test is not appropriate, not described, or incomplete

This scale has been adapted from the Newcastle–Ottawa Quality Assessment Scale for cohort studies to perform quality assessment of cross-sectional studies for the systematic review entitled 'Are Healthcare Workers' Intentions to Vaccinate Related to their Knowledge, Beliefs and Attitudes? A Systematic Review'

**Newcastle–Ottawa Scale – Case–control studies [2]**

Note: A study can be awarded a maximum of one star for each numbered item within the Selection and Exposure categories. A maximum of two stars can be given for Comparability.

**Selection:** (Maximum 4 stars)

1) Is the case definition adequate?
   a) Yes, with independent validation*
   b) Yes, e.g. record linkage or based on self-reports
   c) No description
2) Representativeness of the cases
   a) Consecutive or obviously representative series of cases*
   b) Potential for selection biases or not stated
3) Selection of Controls
   a) Community controls*
   b) Hospital controls
   c) No description
4) Definition of Controls
   a) No history of disease (endpoint) *
   b) No description of source

**Comparability:** (Maximum 2 stars)

1) Comparability of cases and controls on the basis of the design or analysis
   a) Study controls for toothbrushing *
   b) Study controls for other demographic factors*

**Exposure:** (Maximum 3 stars)

1) Ascertainment of exposure
   a) Secure record*
   b) Structured interview where blind to case/control status*
   c) Interview not blinded to case/control status
   d) Written self-report or medical record only
   e) No description
2) The same method of ascertainment for cases and controls
   a) Yes*
   b) No
3) Non-response rate
   a) The same rate for both groups*
   b) Non-respondents described
   c) Different rate and no designation

**Study Quality [3] (case–control study)**

The following are accepted thresholds for converting the Newcastle–Ottawa scales to AHRQ standards (good-, fair-, and poor-quality studies):

- "Good quality: 3 or 4 stars in the selection domain AND 1 or 2 stars in the compatibility domain AND 2 or 3 stars in outcome/exposure domain"

- "Fair quality: 2 stars in the selection domain AND 1 or 2 stars in the comparability domain AND 2 or 3 stars in the outcome/exposure domain"

- "Poor quality: 0 or 1 star in the selection domain OR 0 stars in the comparability domain OR 0 or 1 stars in the outcome/exposure domain"

**Study Quality (cross-sectional study)**

- "Very good quality: 9-10 points"

- "Good quality: 7-8 points"

- "Satisfactory quality: 5-6 points"

- "Unstatisfactory quality: 0-4 points"

**Supplementary Table S1. Risk of bias assessments for cross-sectional studies – Newcastle–Ottawa Scale (adapted version)**

| Source | Selection | | | | Comparability based on design and analysis | Outcome | | Total | Assessment |
|---|---|---|---|---|---|---|---|---|---|
| | Representativeness of the sample | Sample size | Non-respondents | Ascertainment of the exposure | | Assessment of outcome | Statistical test | | |
| Moreira, 2021 [4] | 1 | 1 | 1 | 2 | 1 | 2 | 1 | 9 | Very good |
| Abbass, 2019 [5] | | | 1 | 1 | 2 | 2 | 1 | 6 | Satisfactory |
| Lee, 2019 [6] | 1 | 1 | 1 | 2 | 2 | 2 | 1 | 10 | Very good |
| Lee, 2020 [7] | 1 | 1 | 1 | 2 | 2 | 2 | 1 | 10 | Very good |
| Pujara, 2016 [8] | | | 1 | 1 | 1 | 2 | 1 | 6 | Satisfactory |
| Shimazaki, 2008 [9] | | | 1 | 2 | 2 | 2 | 1 | 8 | Good |
| Al-Zahrani, 2006 [10] | 1 | 1 | 1 | 2 | 1 | 2 | 1 | 9 | Very good |
| Nishida, 2000 [11] | 1 | 1 | 1 | 2 | 1 | 2 | 1 | 9 | Very good |

**Supplementary Table S2. Risk of bias assessment for case–control studies – Newcastle–Ottawa Scale**

| Source | Selection | | | | Comparability based on design and analysis | Outcome | | | Total | Assessment |
|---|---|---|---|---|---|---|---|---|---|---|
| | Is the case definition adequate? | Representativeness of cases | Selection of controls | Definition of controls | | Assessment of exposure | The same method of ascertainment for cases and controls | Non-response rate | | |
| Pulikkotil, 2020 [12] | 1 | | 1 | | 1 | | 1 | 1 | 5 | Fair |

## References


1. Herzog, R.; Álvarez-Pasquin, M.J.; Díaz, C.; Del Barrio, J.L.; Estrada, J.M.; Gil, Á. Are healthcare workers' intentions to vaccinate related to their knowledge, beliefs and attitudes? A systematic review. *BMC public health* **2013**, *13*, 154.
2. Peterson, J.; Welch, V.; Losos, M.; Tugwell, P. The Newcastle-Ottawa scale (NOS) for assessing the quality of nonrandomised studies in meta-analyses. *Ottawa: Ottawa Hospital Research Institute* **2011**.
3. Langan, C.; Sarode, D.P.; Russ, T.C.; Shenkin, S.D.; Carson, A.; Maclullich, A.M. Psychiatric symptomatology after delirium: a systematic review. *Psychogeriatrics* **2017**, *17*, 327-335.
4. Moreira, A.R.O.; Batista, R.F.L.; Ladeira, L.L.C.; Thomaz, E.; Alves, C.M.C.; Saraiva, M.C.; Silva, A.A.M.; Brondani, M.A.; Ribeiro, C.C.C. Higher sugar intake is associated with periodontal disease in adolescents. *Clin Oral Investig* **2021**, *25*, 983-991, doi:10.1007/s00784-020-03387-1.
5. Abbass, M.M.S.; Rady, D.; Radwan, I.A.; El Moshy, S.; AbuBakr, N.; Ramadan, M.; Yussif, N.; Al Jawaldeh, A. The occurrence of periodontal diseases and its correlation with different risk factors among a convenient sample of adult Egyptian population: a cross-sectional study. *F1000Res* **2019**, *8*, 1740, doi:10.12688/f1000research.20310.2.
6. Lee, K.; Kim, J. Dairy food consumption is inversely associated with the prevalence of periodontal disease in Korean adults. *Nutrients* **2019**, *11*, 1035.
7. Lee, J.H.; Lee, S.A.; Kim, H.D. Periodontitis and intake of thiamine, riboflavin and niacin among Korean adults. *Community dentistry and oral epidemiology* **2020**, *48*, 21-31.
8. Pujara, P.; Sharma, N.; Parikh, R.J.; Shah, M.; Parikh, S.; Vadera, V.; Kaur, M.; Makkar, I.; Parmar, M.; Rupakar, P., et al. Effect of westernization on oral health among college students of Udaipur City, India. *Mil Med Res* **2016**, *3*, 32, doi:10.1186/s40779-016-0103-4.
9. Shimazaki, Y.; Shirota, T.; Uchida, K.; Yonemoto, K.; Kiyohara, Y.; Iida, M.; Saito, T.; Yamashita, Y. Intake of dairy products and periodontal disease: the Hisayama Study. *J Periodontol* **2008**, *79*, 131-137, doi:10.1902/jop.2008.070202.
10. Al-Zahrani, M.S. Increased intake of dairy products is related to lower periodontitis prevalence. *J Periodontol* **2006**, *77*, 289-294, doi:10.1902/jop.2006.050082.
11. Nishida, M.; Grossi, S.G.; Dunford, R.G.; Ho, A.W.; Trevisan, M.; Genco, R.J. Dietary vitamin C and the risk for periodontal disease. *Journal of periodontology* **2000**, *71*, 1215-1223.
12. Pulikkotil, S.J.; Nath, S.; Ramachandran, V. Determinants of periodontitis among a rural Indian population: A case control study. *Community dental health* **2020**, *37*, 26-31.
